# Determinants of preconception care among pregnant women in an urban and a rural health facility in Kenya: a qualitative study

**DOI:** 10.1186/s12884-021-04201-w

**Published:** 2021-11-08

**Authors:** J. K. Okemo, D. Kamya, A. M. Mwaniki, M. Temmerman

**Affiliations:** Aga Khan University, Nairobi, Kenya

**Keywords:** Preconception care, Kenya, Urban, Rural, Utilization, Determinants

## Abstract

**Background:**

Preconception care (PCC) is a form of preventive health care that is offered to women and couples before conception, with the aim of improving their health status and mitigating various risk factors that could contribute to poor maternal and child health outcomes. The levels of PCC utilization are still low globally, especially in developing countries and in rural areas. Little is known regarding PCC use in Kenya that could help in addressing this shortfall. This study aimed to qualitatively assess the determinants of PCC in urban and rural settings in Kenya.

**Methods:**

A qualitative approach was employed to assess determinants of PCC using a semi-structured interview guide. The study was conducted from May to October 2017. Selected pregnant women seeking antenatal care (ANC) were recruited by quota sampling, at the Mother and Child Health (MCH) clinics in Aga Khan University Hospital, Nairobi (AKUH, N-urban) and Maragua Level Four Hospital (MLFH-rural). The interviews were thereafter transcribed verbatim and analyzed thematically.

**Findings:**

A total of 26 women were invited, of whom 21 accepted to participate in in-depth interviews (IDIs). Saturation of themes occurred with 13 interviews (7 at AKUH and 6 at MLFH).

Transcription, coding and thematic analysis of the IDIs yielded 12 themes. Eleven of these themes were identified as determinants of PCC. The twelfth theme contained suggested strategies of increasing PCC awareness and utilization, such as using the media, setting up PCC clinics and integrating PCC into other clinics. The dominant themes were awareness about PCC and attitudes towards PCC and pregnancy.

The broad determinants of PCC were similar in urban and rural settings – with a few notable exceptions. For example, in the rural setting, women’s level of education and a pervasive history of poor interactions with healthcare providers were major determinants of PCC.

**Conclusion:**

From this study we conclude that women’s lack of awareness about PCC, in conjunction with attitudes towards PCC and pregnancy impact strongly on its utilization. This lack of awareness could be addressed through health education programs for both the public and for healthcare providers, as well as integrating PCC in the curricula of the later.

**Supplementary Information:**

The online version contains supplementary material available at 10.1186/s12884-021-04201-w.

## Background

Preconception care (PCC) is a set of evidence-based interventions provided to women and couples in the pre-pregnancy period in order to improve their health status and mitigate various risk factors that could contribute to poor maternal and child health outcomes [1–3]. The World Health Organization (WHO) [1, 2, 4] recommended PCC to include the following: reproductive health planning through use of contraception; screening and management of medical, behavioral and social pregnancy risks; health education and promotion; vaccinations and nutritional supplementation.

The level of PCC in developed parts of the world albeit low (a range of 27–39%), are higher than in developing world (a range of 2.7–14%) [5–11]. Several studies conducted in different parts of the world have revealed varying PCC determinants. Some of the barriers to PCC identified in a United States based study [5] included: lack of awareness of the risk factors and their impact on maternal and fetal outcomes by the women, time constraints and insufficient training and knowledge of PCC by the health providers. A study in the United Kingdom [6] revealed poor awareness of PCC among women and health providers as hindrances to PCC, alongside lack of resources and confusion about responsibility for PCC delivery. An Italian study [12] indicated additional determinants of PCC such as considering conception as a natural event, low policy priority by government health agencies and media carelessness. Elsewhere, two separate Ethiopian studies [11, 13] revealed more PCC determinants such as presence of chronic diseases, prior adverse birth outcomes and partner involvement. Even though some of the PCC determinants are shared across different parts of the world, there are contextual differences across the globe that would necessitate tailored strategies.

Although the Ministry of Health in Kenya and the Kenya National Reproductive Health Strategy (KNRHS) of 2009–2015 [14] recommended PCC as one of the six pillars aimed at attaining the fourth and fifth Millennium Development Goals (MDGs) [15], there is paucity of information on how this was to be accomplished. These MDGs have since been replaced by the Sustainable Development Goals (SDGs) [16]. The other pillars included focused antenatal care (ANC), essential obstetrics care, essential newborn care, targeted postpartum care and post-abortion care. The suggested foundation towards attaining these pillars consisted of skilled health attendants, enabling environment, supportive health systems, community, partnerships and male involvement. Of the six pillars, PCC was the only one without clear guidelines and implementation strategies in the country. According to KNRHS, some of the key challenges to overall maternal and neonatal health service delivery were weaknesses in the health sector that negatively affect access to care and the various cultural and socio-economic barriers to skilled care [14].

Differences in the level of other maternal health services in urban and rural Kenya may imply differences in the determinants of PCC [17, 18]. According to the Kenya Demographic Health Survey (KDHS) 2014 [17], the unmet need for contraception is 18% for married women and 27% for sexually active unmarried women and is higher in rural (20%) than urban (13%) areas. Efforts to increase contraceptive prevalence rate (CPR) in Kenya (currently at 62% in urban and 56% in rural) have however been met by challenges such as poverty, religious and cultural beliefs and practices, and weak health systems [15]. The KDHS 2014 [17] revealed that a majority of women in Kenya start their ANC after 4 months of pregnancy with only 19.8% initiating it within the first 4 months. Studies that have looked at the determinants of various maternal health services utilization in Kenya found level of education, marital status, age, employment and accessibility to health facilities as factors affecting utilization [19, 20]. No studies were identified in literature that have looked at PCC determinants in Kenya. Therefore, this study sought to qualitatively assess the determinants of PCC utilization among women in urban and rural settings in Kenya. A qualitative approach would provide a deeper understanding of the contextual determinants of PCC utilization in Kenya. Consequently, this would allow a tailored approach in improving PCC utilization in the country.

## Methods

### Design and study setting

A qualitative approach was used to gain an understanding of the contextual determinants (both enablers and barriers) of PCC in the two study settings through in-depth interviews (IDIs).

The study was conducted at the Aga Khan University Hospital, Nairobi (AKUH, N-urban setting of the study) and Maragua Level Four Hospital (MLFH -rural setting of the study) mother and child health clinics (MCH) clinics. The background information on both study settings have been elaborated in the quantitative study which looked at the level and determinants of PCC in AKUH, N and MLFH [21]. Aga Khan University Hospital, Nairobi is an urban private, tertiary, teaching and referral health facility located in Nairobi County-an all urban county. It is an academic health care centre providing tertiary level healthcare. Maragua Level Four Hospital is a public hospital located about one kilometer from Maragua town in the southern part of Murang’a County. Level four hospitals are the first referral hospitals in Kenya, with both outpatient, inpatient and referral services. They have the following clinical services which are run by either an on-site specialist in the field or a specialist who covers several of the level four hospitals within the region: obstetrics and gynaecology; child health; medicine; surgery and anaesthesia. Murang’a County has a dense rural settlement with 89% of the population living in rural areas and only 11% living in urban centres. Maragua constituency is largely rural. Most of the road linkages within the county are all-weather roads with some of the economic activities including farming, quarrying, forestry and tourism.

### Study participants and recruitment

The study population included pregnant women regardless of their gestational age and ANC visit attending MCH clinics at the two hospitals. The study was conducted from May to October 2017. The participants were eligible if they were aged ≥18 years and were attending their antenatal visit during current pregnancy. The study was carried out among pregnant women attending MCH clinics for ANC in the two hospitals since from the KDHS 2014 report [17, 22, 23], over 97% of pregnant women in the two counties have at least one contact with a skilled health provider. Obtaining study participants from the population of pregnant women attending MCH clinic in both hospitals provided a good representation of the pregnant women population and by extension, reproductive-aged women.

Quota sampling was used to recruit pregnant women, via face to face approach, who took part in the IDIs. Following registration and billing at the front desk, the patients’ files were kept with the triage nurse. The principal investigator, stationed in the triage room, went through these files as they came in to review patient demographics and obstetrics characteristics in order to identify suitable and potential candidates. The participants were stratified (quota sampling) in order to ensure diversity of responses. The stratification was based on age, marital status, education level, occupation, parity and past obstetric history. This resulted in a wider representation of opinions and richer data on the subject of PCC.

### Study tools

The tools used in the study were written in both English and Kiswahili languages. Literacy levels in Kenya as per KDHS 2014 is at 88% (up from 85% in 2008–09) [17]. In addition, we did not come across any willing participant who was left out due to language barrier. Firstly, there was the consent explanation form that contained the following details: introduction of the principal investigator, type of study and how it was going to be conducted, duration of the interviews (given as an average of 20–30 min), potential benefits of the study and the voluntariness to participate. This was followed by the consent form where the participants gave an informed written consent to participate. Lastly, there was the semi-structured interview guide that was used for the IDIs. The questions in the interview guide were developed to reflect the study objectives and literature review and were broadly categorized to address the following: opinions about PCC, content of PCC, risk perception and barriers to PCC. They were thereafter pretested on 8 pregnant women in AKUH, N and MLFH MCH clinics to test the idioms of the languages used, check whether the questions were appropriately framed, inoffensive, clear, easy to understand, able to elicit discussions and address the intended questions for the study. They were thereafter found to be ideal for the study and did not require any adjustments. Thus, a 10 question semi-structured interview guide was used to conduct the IDIs. Of note is that the actual study did not include any of the pregnant women who took part in the pretest.

### Data collection procedures

The study definition of PCC utilization was contact with any health provider before current pregnancy and having discussed about pregnancy planning and preparation. Ethical approval was be obtained from the Aga Khan University, Nairobi Research and Ethics Committee, Ref: 2016/REC-61 (v2) dated 16th February 2017. Permission to conduct the study was also be sought from the hospital administrative committee in MLFH, which has oversight of research and ethics approval at this site. Data collection was carried out at both study sites by the principal investigator. This was done during the waiting period before consultation with the health providers at the respective MCH clinics.

Quota sampling was used to recruit pregnant women, via face to face approach, who took part in the IDIs. The study and its purpose were explained during recruitment and thereafter an informed, written consent to take part in the study was obtained from all the participants. The interviews were conducted in one of the consultation rooms at the MCH in both sites with a “Do not Disturb” sign placed at the door during the interviews to minimize interruptions and uphold privacy and confidentiality of the participants. All the interviews were audio recorded and lasted an average of 13 min.

The gold standard for determining sample size in qualitative studies is ‘saturation’ where the investigator continues to seek information until no significant new information or themes are anticipated to arise from further interviews [24]. An estimated number of interviews to be conducted was 15 in each site, derived from literature review where most of the PCC qualitative studies involving interviews had a sample size of 12 to 27 participants [12, 24]. However, the interviews in this study were conducted to saturation, which was attained with 7 and 6 interviews in AKUH, N and MLFH respectively. Further, 4 interviews were conducted in each of the study sites to confirm saturation.

**Fig. 1:**
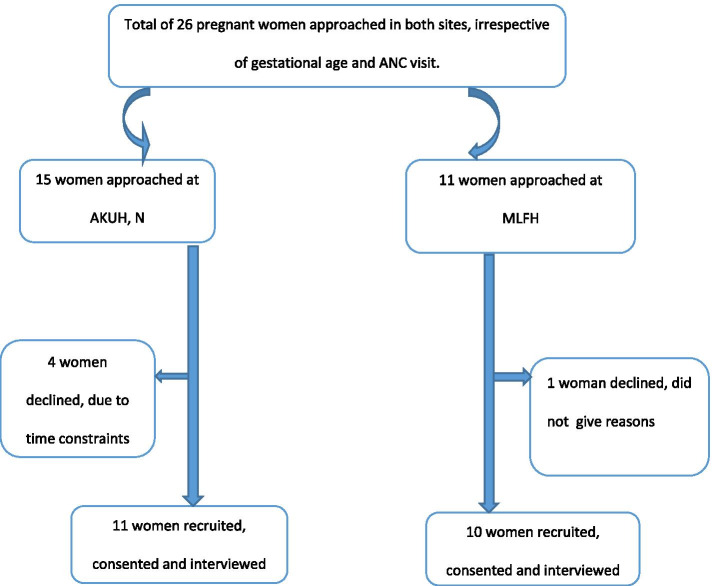
Flow of participants

Figure 1 below (*appearing after the references section on page 27)* shows the study flow diagram.

### Data analysis

Each interview was immediately transcribed verbatim into Microsoft word by JKO. Each IDI transcript was read and reread for data familiarization and thereafter, inductive coding was done line by line by JKO and two other independent coders. Coding was done by selection of significant/relevant sections from the participants’ statements as they emerged. The significant/relevant sections were as follows: statements that the participants stated as key or were repeated severally, unexpected information, information that was recurrent in literature or any other statement that the coders deemed important or relevant to the study. Analyst triangulation was employed by having two individuals-not co-authors, (CK-a research scientist with experience in qualitative research and SN-a social scientist) separately listen to the audio recordings, confirm the transcripts and identify codes. The three discussed their codes, modified or identified new codes where discrepancies arose and developed an agreed upon list of codes. Indexing references were noted at the margins of each transcript by descriptive textual system based on index headings. This helped in identification of patterns and associations and the context in which they arose. Data from the original transcripts were then organized into charts with headings and sub-headings and constructed into a thematic framework [25]. Thereafter, subthemes and themes were formed by looking for linkages between categories through identification of patterns, frequencies of occurrence, differences and making judgements of relationships by JKO, CK, SN and DK. Saturation was achieved with 7 interviews in AKUH, N and 6 in MLFH. Further 4 interviews were conducted in each site to ensure no new information was subsequently emerging. Mapping and data interpretation were thereafter carried out by JKO, DK, MT and MM through defining key concepts, mapping the range and diversity of responses and seeking explanations internally within the data.

## Findings

### Study participants

Table 1 below *(appearing after the references section on page 27–28)* summarizes the characteristics of the participants and shows that the women interviewed were comparable in age, parity and PCC utilization across both sites. Whereas all the participants at AKUH, N were university graduates with a majority being professionals and married, the participants in MLFH were more diverse in terms of their marital status, education levels and occupation.

**Table 1:** Socio-demographic characteristics of study participants

Site	AKUH, N	MLFH
Age in years	Mean = 31.2 Median = 30 Range = 26–39	Mean = 28.8 Median = 29.5 Range = 22 to 37
Marital status	Married 10/11 (90.9%) Widowed 1/11 (9.1%)	Married 7/10 (70%) Single 2/10 (20%) Divorced 1/10 (10%)
Level of education	University 11/11 (100%)	Primary school 1/10 (10%) Secondary school 3/10 (30%) College 3/10 (30%) University 3/10 (30%)
Occupation	Professionals 10/11 (90.9%) Unemployed 1/11 (9.1%)	Professionals 3/10 (30%) Business 3/10 (30%) Farmer 2/10 (20%) Student 1/10 (10%) Unemployed 1/10 (10%)
Median Parity	1 + 0	1 + 0
Fraction who received PCC	Yes 5/11 (45.5%)	Yes 3/10 (30.0%)

### Preconception care determinants

Twelve themes emerged from the data as illustrated in Table 2 below (*appearing after references section below on pages 28–32*), eleven of which were identified as PCC determinants and the twelfth theme captured the strategies for increasing PCC awareness and utilization. The key determinants of PCC from this study included: awareness about PCC, availability and accessibility of PCC, responsibility for PCC and attitudes towards pregnancy and PCC in general. Other PCC determinants that emerged were previous interactions with health professionals as well as social influences, health seeking behaviour, health concerns and level of education. The majority of the PCC determinants were comparable in the two study sites with the following exceptions: attitude towards PCC and previous interactions with health professionals (both were more in the rural setting), and social influences (more in the urban setting).

**Table 2:** Summary of the twelve themes, with an illustrative quote from the IDIs

Main themes	Sub themes	Quote	Ref (source)
1. Awareness about PCC	-Lack of/ awareness about PCC -Unawareness about timing and content of PCC -Misconceptions on PCC -Awareness about risk factors to pregnancy and mitigations	“I did not know there was even such a thing! I have never heard of it. No, people are not aware about it. Like myself I have heard it for the very first time with you.”	PCC 009, AKUH, N
2. Availability of PCC	Un/availability of PCC	“I think it’s important it’s just that I am not sure it’s usually available. If I knew how to access it I would probably do it.”	PCC 009, AKUH, N.
3. Accessibility of PCC	-Financial constraints -Time and occupational constraints -Area of residence -Distance to health facility	“If I can’t afford it, I would ignore it. Sometimes you even convince yourself that you don’t actually need this care cos you don’t have money for it”	PCC 004, AKUH, N
4. Responsibility for PCC	-Lack of consensus on whose it was to initiate PCC -Role of health providers	“I think the responsibility for preconception care goes both ways. The greater responsibility lying on the individual, the patient but I think the initial part at least starts with the health care professional’s side.”	PCC 065, MLFH
5. Attitudes towards pregnancy in general	-Lack of pregnancy planning -Downplaying pregnancy and taking it as a natural phenomenon -Feeling one is well versed on pregnancy and childbirth -Prior good pregnancy outcomes -Blaming witchcraft -Parity -Prior bad pregnancy outcomes and fear	“We had counseling and doctor reviews after the stillbirth of my second born, just to be ready for the next pregnancy. Before that I had never bothered to seek care before getting pregnant. I always assumed I will have a smooth pregnancy and bouncing babies. After all people get pregnant all the time, even without planning and everything goes just fine.”	PCC 071, AKUH, N
6. Attitude towards PCC	-Dismissing importance of PCC -Personal preferences -Personal beliefs, culture and traditions. -Skepticism about PCC -Understanding importance of PCC -Desire for more information on pregnancy and preparation -Absolution from blame -Procrastination	“If you want a healthy baby you must take care of yourself first and rectify the things in your power to change. Sometimes things can go wrong like when you get a miscarriage or something goes wrong with your baby it is better if you know you did everything that you could have done to make things okay. Also, some people just want to be healthy during pregnancy and carry pregnancy to term without any health issues.”	PCC 020, MLFH
**Minor themes**			
7. Previous interactions with health professionals	-Communication skills and nature of relationships with health providers -Fear, confidentiality and stigmatization concerns -Unmet expectations and frustrations in the past	“We have been telling ourselves that doctors are harsh and bad. When you go to the hospital they can harass and embarrass you.”	PCC 036, MLFH
8. Social influences	-Role of partner, traditional birth attendants, friends and family -Social media and internet	“I personally advocate for PCC. So I have been talking to a few people who I know are getting married, I have been advising them, make sure if you want to get pregnant start at an early age, quit alcohol, be fit, you know prepare your body.”	PCC 071, AKUH, N
9. Health seeking behaviour	-Health checkups and ascertaining health status -Seeking information on what pregnancy entails	“I need to know what to expect, be aware of potential pregnancy challenges and complications and how to overcome them. Maybe most of them can actually be reduced with good planning and preparation.”	PCC 020, MLFH
10. Health concerns	-Coexisting medical and genetic conditions -Fertility concerns -Family planning concerns	“Well some women have conditions that may affect the baby or their health may be worsened by the pregnancy like heart condition so they might want to talk to a doctor before they conceive.”	PCC 051, AKUH, N
11. Level of education	-Level of education	“Those who are learned go to the hospital to prepare for pregnancy. Those who are uneducated don’t understand PCC”	PCC 019, MLFH
12. Strategies for increasing PCC awareness and utilization	-Public education using print and broadcast media -Availing PCC in institutions like churches and learning institutions -Initiating PCC services through clinics -Government initiatives like free PCC services.	“A good point to catch people is at the family planning clinic. Getting the PCC information out there would help even by raising awareness through the social media, just get the conversation happening.”	PCC 009, AKUH, N

### Awareness about preconception care

Most (8 out of 11) of the pregnant women in AKUH, N and all of those from MLFH highlighted awareness about PCC as a major determinant of utilization. Lack of awareness about PCC was a barrier, whereas knowledge about it was a promoter of its use. Some pregnant women from MLFH felt that the people in urban areas knew more about PCC than them.

*“I didn’t know there was even such a thing! I have never heard of it. No, people are not aware about it. Like myself I have heard it for the very first time with you.”* (PCC 009, AKUH, N).

*“No, very few people are aware. Those who know are those who have maybe lived in town or are educated. But if you go to the villages like here, many women don’t know.”* (PCC 036, MLFH).

### Availability of preconception care

Even though some women knew about PCC, unavailability of the care locally was highlighted as a barrier to utilization. Some of them felt that PCC clinics and services were generally unavailable in the country, or if available, information on where to obtain the services was lacking.

*“I think it’s important it’s just that I am not sure it’s usually available. If I knew how to access it I would probably do it.”* (PCC 009, AKUH, N).

### Accessibility of preconception care

The crucial hindrances to PCC under this theme were: distance, finances, time and occupational constraints. Some respondents felt that health care was generally expensive, even with insurance. Therefore, those who deemed themselves as healthy would rather use that money on other competing needs. Long queues at the health facilities, strenuous working hours and lack of time keeping for appointments were also highlighted by a number of respondents as reasonable obstacles to accessing PCC. One participant from MLFH raised the issue of distance to health facilities as a stumbling block.

*“If I can’t afford it, I would even ignore it. Sometimes you even convince yourself that you don’t actually need this care cos you don’t have money for it.”* (PCC 004, AKUH, N).

*“Time is also an issue. People don’t want to leave their farms and businesses to come queue for treatment.”* (PCC 020, MLFH).

### Responsibility for preconception care

A majority of the respondents from both sites expressed confusion about whose responsibility it was to initiate PCC. Whereas some felt it was squarely a woman’s responsibility, others thought it was solely the health providers’ since they were more knowledgeable about the subject, while others felt it involved everyone in the community. Respondents felt that health providers played a role in both hindering and promoting care through the nature of care and type of information they gave to the patients.

*“I think the responsibility for preconception care goes both ways. The greater responsibility lying on the individual, the patient but I think the initial part at least starts with the health care professional’s side.”* (PCC 009, AKUH, N).

### Attitude towards pregnancy in general

Respondents’ attitudes towards pregnancy varied widely, some talking about their lack of pregnancy planning, some downplaying their pregnancies and taking pregnancy as a natural phenomenon which cannot be enhanced or prepared for, and a few blaming witchcraft for bad pregnancy outcomes. A few pregnant women mentioned increasing parity and having prior good outcomes as valid reasons not to use PCC. Conversely, those who had a planned or first pregnancy or those who had prior bad obstetric outcomes promoted use of PCC.

*“Sometimes you get pregnant without planning. But if you can plan then I think PCC use is possible.*” (PCC 037, MLFH).

*“We had counseling and doctor reviews after the stillbirth of my second born, just to be ready for the next pregnancy. Before that I had never bothered to seek care before getting pregnant. I always assumed I will have a smooth pregnancy and bouncing babies. After all people get pregnant all the time, even without planning and everything goes just fine.”* (PCC 071, AKUH, N).

### Attitude towards preconception care

Some respondents felt that they were well informed about what pregnancy and its preparation entailed hence, they did not see the need for PCC, while some out rightly dismissed the importance of PCC or kept procrastinating their PCC visits. Some, especially those from MLFH, held personal beliefs, myths, culture and traditions that contradicted use of PCC. A few respondents had varied perceptions that hindered PCC use such as, considering themselves healthy enough to require PCC or being skeptic about benefits of PCC. Conversely, some women sought PCC to absolve themselves from blame in case of bad obstetric outcomes and some had a desire for a healthy baby and good maternal health which drove them to PCC utilization.

*“I have heard of this care before, but from my understanding it doesn’t apply to Africans. I don’t think there is anyone in Africa who does that, you know, seeking health care before you get pregnant! It is a Western culture thing.”* (PCC 065, MLFH).

*“If you want a healthy baby you must take care of yourself first and rectify the things in your power to change. Sometimes things can go wrong like when you get a miscarriage or something goes wrong with your baby and it is better if you know you did everything that you could have done to make things okay. Also, some people just want to be healthy during pregnancy and carry pregnancy to term without any health issues.”* (PCC 020, MLFH).

### Poor previous interactions with health care professionals

A few respondents from MLFH expressed fear and concerns of confidentiality and stigmatization during their interactions with health providers such as those living with HIV/AIDS. Some, having experienced unmet expectations and frustrations during previous consultations with health providers, opted not to seek PCC*.*

*“Other people their HIV status makes them not to seek PCC. Many are afraid (laughs). Maybe they are not comfortable, someone feels she will be judged by nurses or her status will be known in the area.”* (PCC 019, MLFH).

### Social influences

A woman’s social influences including traditional birth attendants, partners, friends and family played a role in PCC utilization. Some of them provided the social or financial support that women needed, while others advocated for PCC and urged them to seek it. However, there were those who barred them from seeking the care or urged them to seek help elsewhere.

*“If I get support, especially like my husband giving financial support, advice and emotional support or I have someone who guides me, I think I can do it (PCC).”* (PCC 036, MLFH).

*“I personally advocate for PCC. So I have been talking to a few people who I know are getting married, I have been advising them, make sure if you want to get pregnant start at an early age, quit alcohol, be fit, you know prepare your body.”* (PCC 071, AKUH, N).

### Positive health seeking behaviour

Health checkups and seeking information about what pregnancy entailed were shared by a few respondents as good reasons for seeking PCC. Some respondents felt it was prudent for one to see a health professional before conception to check their health status and identify any underlying risks that could negatively impact the pregnancy. Others were more interested in gathering information on what to expect during pregnancy, how to best prepare for it, possible complications and how to avoid those complications.

*“For me I need to know what to expect, be aware of potential pregnancy challenges and complications and how to overcome them. Maybe most of them can actually be reduced with good planning and preparation.”* (PCC 020, MLFH).

### Health concerns

A few respondents felt that having a genetic or preexisting medical condition was important enough to warrant seeking PCC services, with the aim of optimizing health status before and during pregnancy. Those with fertility and family planning concerns such as delay in conception were also inclined to seek PCC to achieve conception and to ensure subsequent good pregnancy outcomes.

*“Well some women have conditions that may affect the baby or their health may be worsened by the pregnancy like heart condition so they might want to talk to a doctor before they conceive.”* (PCC 051, AKUH, N).

### Level of education

A participant from the rural setting highlighted education as crucial in equipping women with knowledge that helps them to understand the importance of PCC.

*“Those who are learned who go to the hospital to prepare for pregnancy. Those who are uneducated don’t understand PCC”* (PCC 019, MLFH).

### Strategies for increasing PCC awareness and utilization

Various suggestions were fronted by respondents from both study sites on how to increase PCC awareness and utilization in Kenya. Suggestions for increasing PCC awareness ranged from use of print media such as postas, billboards and flyers in frequented places like the accident and emergency area to use of broadcast media (including in vernacular languages) and internet media. Other suggestions for increasing PCC awareness included use of social forums such as bridal showers, churches and learning institutions (including high schools) and carrying out national PCC campaigns.

Government-led initiatives such as incorporating PCC in the free maternity program and providing PCC during other hospital visits such as family planning and postnatal were shared as reasonable ways of increasing PCC utilization. Other plausible ways of increasing PCC utilization included having community outreach programs, encouraging partner involvement, having free PCC services months and initiating “mum to be” clinics.

*“Availing PCC information. A good point to catch people is at the family planning clinic. Getting the PCC information out there would help even by raising awareness through the social media, just get the conversation happening.”* (PCC AKUH 009).

## Discussion

The key determinants of PCC from this study included: awareness about PCC, availability and accessibility of PCC, responsibility for PCC and attitudes towards pregnancy and PCC in general. Other PCC determinants that emerged were previous interactions with health professionals as well as social influences, health seeking behaviour, health concerns and level of education. Women’s lack of knowledge about PCC did not only encompass a lack of awareness of the existence of PCC but also awareness of the content and timing of PCC. The latter two guided women to know whether and when they need PCC services [2, 3, 26]. Awareness about the risk factors that may lead to poor pregnancy outcomes, and the existence of risk mitigation measures through PCC emerged as a key factor in determining if women were willing to use PCC. Some women in this study with pregnancy risk factors, though aware of PCC, may have opted not to use PCC if they felt that there was no way of mitigating the risk in order to better pregnancy outcomes. This key finding is comparable to findings in studies by Asresu et al. [13] and Demisse et al. [11] in Ethiopia and an Italian study by Bortolus et al. [12] which also found awareness about PCC as a key determinant. Addressing lack of awareness therefore, should not only entail making known its existence but also making the public aware of potential pregnancy risk factors and the existence of the evidence based interventions that better pregnancy outcomes.

It is possible to postulate that there would be a difference between the PCC uptake in the rural and urban settings here, because of differences in access, educational levels and availability of resources [17]. Although some of the respondents from the rural setting felt that their urban counterparts were more aware of PCC due to their higher education levels and exposure, a majority of the urban women were equally unaware of PCC. Thus being educated did not necessarily translate to being aware of PCC. It is possible that the women from the rural setting assume that higher education is associated with knowledge seeking behavior which would spill over to gaining knowledge beyond one’s area of training. Another possible assumption is that higher education is associated with higher income which in turn buys access to qualified medical advice.

Another instructive example of determinants revealed in this study include lack of awareness about the content of PCC among health providers. This chimes with several studies in literature which found PCC awareness to be an issue not only among women, but also among health providers [6, 27–29]. Therefore, if PCC is to be effectively utilized, a key national health promotion target should be not only be to increase PCC awareness of the public but also of (primary) health care workers and women of childbearing age. Some enlightening ideas from the participants in this study on the most effective ways of increasing PCC awareness includes use of broadcast and print media both of which are widely available in Kenya [17], and equipping all health providers with the same message on PCC concept. As an adjunct goal, health education programs should aim to clear the existing misconceptions about the care. As an example, some women believed PCC aims to give supplements to help with conception and it is therefore only for those with fertility problems.

A majority of the women in both study sites agreed that PCC was a very important form of primary health care. However, due to its unavailability and various accessibility challenges, it was unreachable. Financial and time constraints were underlined as major players in limiting accessibility of PCC to those who desired it both in the urban and rural settings. Due to a myriad other competing financial demands, PCC was sacrificed over more pressing needs. A few women went as far as convincing themselves that they were too healthy to require PCC, simply because it was beyond their reach. Due to the demanding nature of some vocations, poor time keeping and long queues during hospital visits, appointments for PCC consultations could not be honored. Unavailability of PCC was uniformly spread in both study settings. Whereas the people in the urban setting generally have more financial muscle, the issue of time constraints due to hours spent in traffic jam, stringent work hours and long queues in hospitals are critical impediments. On the other, their rural counterparts’ challenge lies not only in the amount of time spent looking for daily bread and butter which wins over PCC, but also in the limited finances. Local studies have demonstrated that similar hindrances face other forms of maternal health services in Kenya [19, 20]. Same barriers to PCC care uptake were also found in other studies in different parts of the world [27, 30–32]. As suggested by a few women, incorporating PCC in the free maternity care program in Kenya and initiating distinct PCC clinics in health care facilities can help to address these barriers.

Participants from both study sites had split opinions on whose responsibility it was to initiate PCC. This means no one in particular is responsible for initiating the care, and could be attributed to lack of guidelines and strategies with regards to PCC delivery in Kenya. This results in a situation where the women who may be most in need of PCC cannot access it – as they rely on healthcare providers to initiate them into the care. This reflects the findings of several studies in literature which also revealed a lack of guidelines on PCC responsibility [5, 6, 12, 27, 30]. Further, studies done among health providers also revealed a lack of consensus on this [27, 30, 33]. Public education to enable understanding of individual responsibilities and clear role definitions would clear the existing confusion. As proposed by some of the participants, incorporating PCC as a must have service during other hospital visits for all reproductive aged women would help health providers assume the responsibility of offering it to their patients.

Respondents from both sites recognized that their attitudes towards pregnancy and PCC, such as pregnancy planning, are important determinants of PCC utilization. Since some women believe that pregnancy is a natural phenomenon that is dictated by higher powers, PCC was seen as less relevant, as they believe one can never really plan or prepare for a pregnancy. Studies elsewhere in the literature reveal that some women believe that it was beyond their power to plan a pregnancy [27, 28, 30–34].. Inability to plan for a pregnancy goes hand in hand with inability to seek PCC. The CPR level in Kenya is at 62% in urban and 56% in rural areas [15], and this may potentially augment the problem of PCC utilization. A few rural women held beliefs that PCC is a Western phenomenon, which makes it irrelevant to Africans. Others’ blame of witchcraft for poor obstetric outcomes shows that some traditional beliefs and practices may get in the way of effective PCC use. Use of trained, older women in the communities and community health workers who can identify with and reach the local women, is one way to address these barriers.

The study also explored the factors that enable or promote PCC use, which were prior poor obstetric outcomes and the need for information about better pregnancy outcomes in women with underlying health conditions. Conversely, good obstetric outcomes in the past led others to believe that pregnancies are smooth, natural phenomenon that did not require any enhancement or interventions. In addition, some parous women felt more knowledgeable and experienced in matters pregnancy such that they felt they did not require any PCC input. This is a fascinating finding as one would expect parous women to be more aware of how dynamic and unpredictable pregnancy and delivery can be, and as a result embrace PCC more. Women therefore need to be educated about the content of PCC and the fact that a woman’s risks are dynamic which doesn’t always guarantee perfect outcomes in view of good outcomes in the past.

The quest for information about what pregnancy entails and how best to prepare for it or to ascertain one’s health status prior to conception also drives others to seek PCC. Further, some receive PCC in view of either the presence of underlying health problems such as chronic and genetic diseases or infertility concerns. It is therefore possible to purport that both among the health professionals and the public, there are some individuals who are knowledgeable, understand the importance of and embrace PCC. This aligns with the findings in studies done in Ethiopia [13], Nigeria [9] and United Kingdom [6, 33]. This comes in handy because social influences such as family, friends and social media play a remarkable role in PCC uptake. Therefore, public education on the concept of PCC to ensure that correct information is in circulation cannot be overemphasized. On the other hand, poor prior interactions with health providers serves as an impediment to not only seeking care, but to also accepting and implementing recommendations made thereafter. This, interestingly, was a dominant theme among the rural women and is echoed by findings from the Northern Nigeria study by Idris (semi-urban setting) [7]. It seems self-evident that being poorly treated does not encourage patients to engage health providers. From personal observations, the submissive culture of some rural communities in Kenya towards people of authority places them in the vulnerable position of receiving instructions and admonition quietly and without questioning or demanding respect. The patient population in the rural parts of Kenya have less financial power, access and influence. Health providers play a crucial role in the delivery of PCC and therefore, communication training and feedback among health providers can be employed as an effort to address this barrier.

### Strengths

This study looked at the determinants of PCC in both urban and rural settings, as well as in private and public hospitals, in a single study. The comparison of the attitudes and experiences of women in rural and urban centres of care is a strength as it allows comparison of women who have different levels of finances and access to PCC, and varied sociodemographic characteristics.

Use of qualitative method allowed us to delve deeper into the contextual factors affecting PCC in Kenya. It also provided additionally solutions to the barriers of PCC from the patients’ perspective.

### Weaknesses

The weakness of this study was the homogeneity of the women from AKUH, N (all had tertiary education) and therefore, these findings may not apply to urban women with differing socio-demographic characteristics like those from the slums hence, a separate study in low resource urban areas may be helpful. Another weakness of this study was the short duration of the IDIs (an average of 13 min) and unavailability of field notes. The fact that we explored PCC determinants on pregnant women and not during the preconception period is also a noteworthy weakness of this study.

## Conclusion

From this study it is possible to conclude that women’s lack of awareness about PCC, in conjunction with lack of availability and access to PCC impact strongly on its utilization in both urban and rural settings. This lack of awareness could be addressed through health education programs for both the public and for healthcare providers, as well as integrating PCC in the curricula of the later. Women’s social influences, educational level and attitudes to PCC and pregnancy were also identified as key determinants of PCC utilization in Kenya. To mitigate some of these, health agencies can train and empower community health workers and traditional birth attendants on PCC concept.

## Supplementary Information

**Additional file 1.**

## Data Availability

The data that support the findings of this study are available from AKUH, N but restrictions apply to the availability of these data, which were used under license for the current study, and so are not publicly available. Data are however available from the authors upon reasonable request and with permission of AKUH, N.

## Abbreviations

PCC: Preconception care
ANC: Antenatal care
MCH: Mother and child health clinic
AKUH, N: Aga Khan University Hospital Nairobi
MLFH: Maragua Level Four Hospital
IDIs: In-depth interviews
W H O: World Health Organization
KNRHS: Kenya National Reproductive Health Strategy
MDGs: Millennium Development Goals
KDHS: Kenya Demographic Health Survey
CPR: Contraceptive Prevalence Rate
HIV: Human Immunodeficiency Virus
