# Supplementary material for: Determinants of preconception care among pregnant women in an urban and a rural health facility in Kenya: a qualitative study
Source: BMC Pregnancy Childbirth. 2021 Nov 8;21:752. doi: 10.1186/s12884-021-04201-w (PMC8573977; doi:10.1186/s12884-021-04201-w)
Supplement: Supplementary file 1 — Additional file 1. [file 12884_2021_4201_MOESM1_ESM.docx]

**SEMI-STRUCTURED INTERVIEW GUIDE**

My name is Dr Joan Okemo, a postgraduate student in Aga Khan University. Thank you for agreeing to participate in this research, we will be discussing about pre-pregnancy care, an issue that is important to all of us. Please feel free to give your opinions openly. Anonymity and confidentiality will be maintained. I would like to tape record the whole interview because I will need to refer back to it when am writing my report later. Please let me know if you are comfortable with this arrangement. The session will take about 20-30 minutes. If this arrangement is okay with you, I would like you to give a written consent to participate in this interview.…. (*Give a consent form for the participant to read, understand and sign it at this point*)………

To start us off, I would like you to briefly share about your pregnancy experience. How has your pregnancy experience been so far?

**Opinions about PCC**

1. What are your thoughts about seeking medical care from any health care professional about pregnancy preparation before a woman gets pregnant? Is this something that you can do? (Explore reasons of why and why not)

2. What are some of the ways you can suggest that could change the level of awareness about health care services before pregnancy in this area?

**Content of PCC.**

3. According to you, what are some of the things that women can do before getting pregnant in order to have a healthy pregnancy and healthy baby? (Probe to find out why)

4. From the questionnaire you filled, you mentioned that you feel your pregnancy happened at the right time (not quite at the right time/wrong time) – refer to questionnaire. If you don’t mind my asking, if you had time to plan and prepare for your pregnancy, is there anything in particular that you would have liked to do? (Probe to find out what and why)

**Risk perception**

5. Do you think there are any behaviors and health issues that might have a potential effect on mothers and their babies during pregnancy? (Probe to find out what and how they would affect)…

6. If you don’t mind sharing with me, do you personally have any of the health issues or behaviors that you have just mentioned which could affect your pregnancy? (If not willing to share, don’t press it….)

**Barriers to care**

7. It seems to me that you were well prepared (not so well prepared) for this pregnancy. Can you think of some reasons why this may be the case?

8. According to you, what are some of the reasons why women seek medical care prior to getting pregnant? Can you think of some reasons why women do not seek medical care prior to getting pregnant?

9. When it comes to this type of health care, whose initiative do you think it **should** be?

Health systems and policies

10. Do you think the government/ministry of health has any role when it comes to pre-pregnancy care? (Probe to find out which roles)

*Thank you so much for your time and your participation in this discussion. All the information you have shared with me will remain confidential and anonymous, it cannot be traced back to you. Have a good day and I wish you the very best in your pregnancy.*

CONSENT EXPLANATION FORM

Dear study participant,

My name is Dr Joan Okemo. I am a female postgraduate student in obstetrics and gynecology at Aga Khan University Hospital, Nairobi. As part of my training, I am carrying out this research project.

I am conducting a study that aims to identify factors that influence usage of pre-pregnancy care, which means seeking and using any form of medical care before getting pregnant as a way of preparing for the pregnancy. When women are planning pregnancy, they may or may not do some things as part of preparation for pregnancy.

Participation in this study entails filling a form that will contain questions routinely asked in health visits about your socio-demographic background. Afterwards, you will be requested to participate in a one on one interview with me to share your insights and opinions about this topic. The interview will take 20-30 minutes and it will be tape recorded. The interview will not prolong your antenatal visit unnecessarily, since it will be conducted during your waiting time.

You may or you may not choose to participate in this study. You are free to opt out of the study at any point in time and this will not affect your antenatal follow up with us. All the information you share during this study will be private and confidential. Your names and contacts will not appear in any of the documents. The information you give here will only be accessible to the lead investigator (myself).

You are free to choose whether or not to participate in this study. This decision will not affect the standard of care that you receive thereafter. There will be no remuneration for participation in this study. There will be no penalties for refusal to participate in this study.

The results obtained from this study will give us information that will eventually help us know how to improve usage of this form of care in our population.

Your participation in this study is highly appreciated.

For further questions and concerns about this, please feel free to contact the lead investigator (myself):

Dr Joan Okemo

Phone number +254 727 36 00 65

Email joan.okemo@aku.edu

Or

Dr Mukaindo Mwaniki

Phone number +254 721 379 862

In case of ethical issues contact the Ethics Review Committee Secretariat at Aga Khan University, P.O Box 30270-00100, Nairobi. Tel 0203662148.

Email research.supportea@aku.edu

CONSENT FORM

I ……………………………………………………………………………………………………………………..have read the consent information form, I have fully understood what is required of me and what the study entails. I hereby consent to take part in this study.

Signature …………………………………………………………………………

Date…………………………………………….

Name of researcher…………………………………………………………….

Signature …………………………………………………………………………..

Date…………………………………………….
